# Reversed Intestinal Rotation Presented as Bowel Obstruction in a Pregnant Woman

**DOI:** 10.1155/2015/870437

**Published:** 2015-05-17

**Authors:** David Aranovich, Ilan Schrier

**Affiliations:** Department of Surgery, Rabin Medical Center, Beilinson Hospital, 39 Jabotinsky Street, 49100 Petah Tikva, Israel

## Abstract

A rare case of complete large bowel obstruction in a pregnant woman, without previous surgical history, due to previously undiagnosed reversed intestinal rotation is presented. The young woman was admitted with progressive nausea and vomiting which did not respond to conventional therapy. Her plain abdominal film revealed signs of small bowel obstruction. On laparotomy, her transverse colon was found to be located beneath the root of small bowel mesentery and completely obstructed by congenital fibrous bands. Postoperative recovery was unremarkable. Surgery for this unusual developmental anomaly is discussed.

## 1. Background

Nausea and vomiting are common in pregnancy. Most of these patients achieve symptomatic control with medical management. Since bowel obstruction is infrequent during pregnancy it may be masked by more commonly occurring emesis or hyperemesis gravidarum. We describe a case of a young woman who presented in second trimester of pregnancy with progressively worsening nausea, vomiting, and abdominal distention. She was diagnosed with a small bowel obstruction, and, upon laparotomy, the transverse colon was fond behind the root of small bowel mesentery, completely obstructed by fibrous bands. Recognizing this reverse rotation and surgical treatment led to successful outcome of the patient and her pregnancy. By reporting this case, we emphasize the role of early recognition of bowel obstruction in a pregnant patient and the role of uncommon developmental anomalies and surgical treatment of reverse intestinal rotation.

## 2. Case Presentation

**Figure 1:**
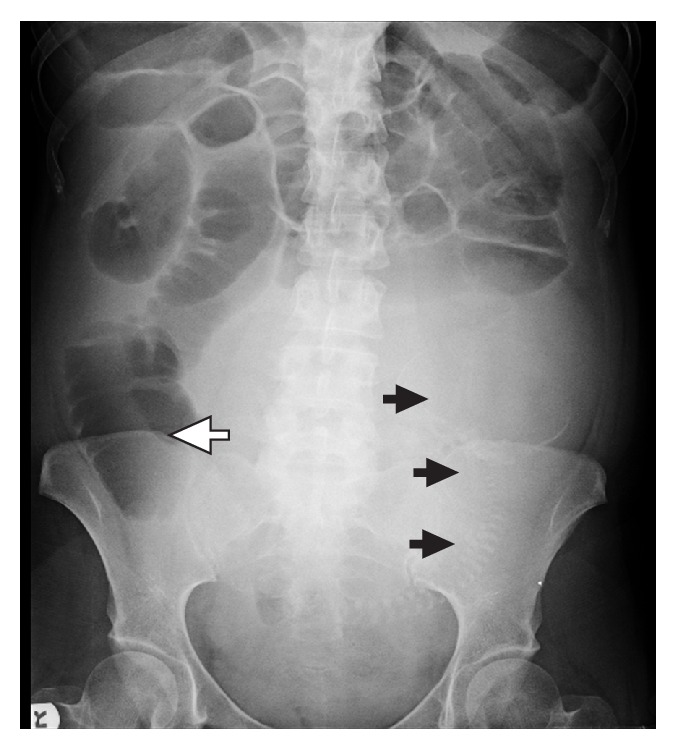
Preoperative abdominal X-ray film. Dilated loops of small abdomen in the upper abdomen. Dilated cecum or massively dilated small bowel loop in right lower abdomen (hollow arrow). Fetal bony silhouette is outlined with solid arrows.

A 27-year-old primigravida at 25th week of gestation was admitted to the OBGYN Department of Rabin Medical Center, Israel, with nausea, vomiting, and abdominal pain. Her past medical and surgical history were unremarkable. During the past three weeks, she complained of nausea and vomiting, which were attributed to hyperemesis gravidarum. During this period, she was seen several times in the emergency room and treated symptomatically with intravenous fluids and antiemetics. On physical examination, she was afebrile, with stable vital signs, frequently vomiting jejunal contents. Her abdomen was compatible with gestation age, but distended and tense. She did not pass flatus for three days. Plain abdominal X-ray film showed distended small bowel loops in the upper abdomen and gasless lower abdomen with fetal bony silhouette (Figure 1). After nasogastric decompression and appropriate fluid resuscitation, she was taken for exploratory laparotomy. The decision to operate was prompted by history of progressive worsening of her symptoms for three weeks, large quantity of jejunal-type fecal vomitus, large volume nasogastric effluent, and clinical and radiological picture of high grade bowel obstruction.

**Figure 2:**
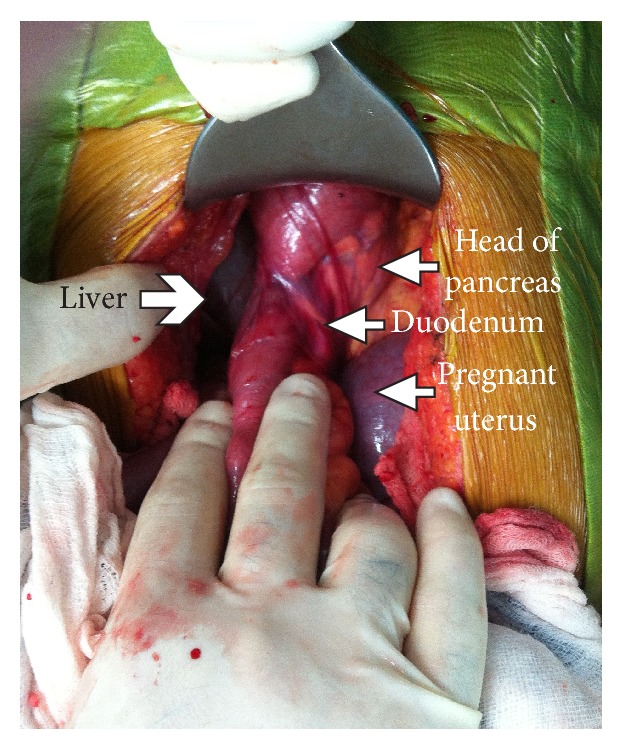
Operative photograph. Duodenum is located on the right side of the mesenteric root and extends as a direct continuation of the antrum, without being curved behind mesenteric axis.

**Figure 3:**
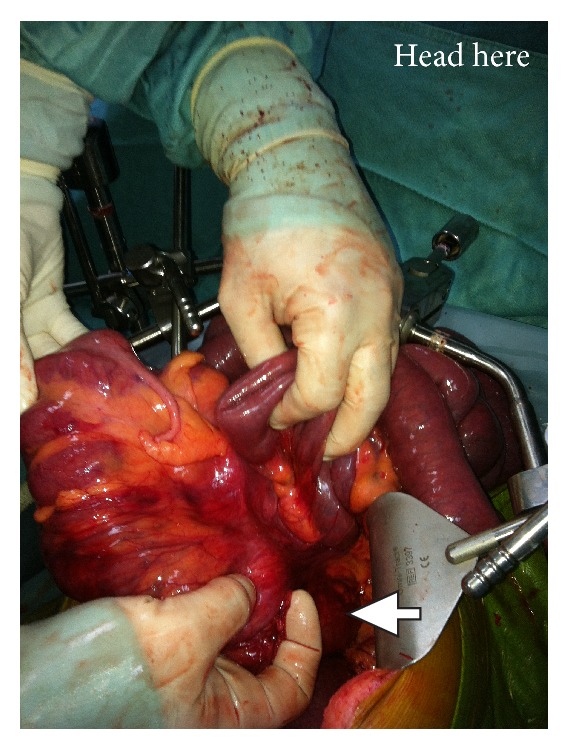
Operative photograph, after releasing obstructive bands. The ileocecal complex and the entire right colon are upside down. The transverse colon passes behind the mesenteric root. Note: the surgeon's index finger indicates the point of obstruction (arrow).

**Figure 4:**
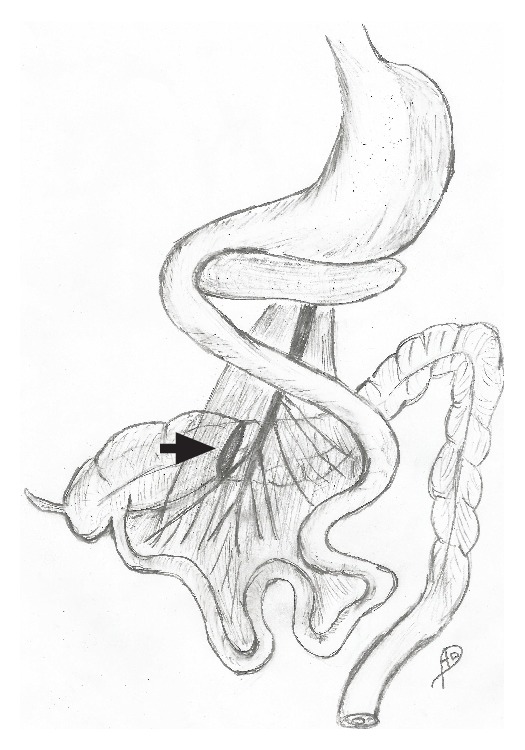
Illustration of the anatomical situation. The entire colon failed to move anteriorly to superior mesenteric axis during embryonic life. The transverse colon is positioned behind the root of mesentery in a rertroarterial position. The site of obstruction is marked with an arrow.

At laparotomy, the duodenum was situated on the right of midline without being curved behind the mesenteric vessels (Figure 2). There was no gastrocolic omentum present and the duodenum was completely exposed in the upper abdomen. The nonfixed cecum was situated under the liver. There was no hepatic flexure and the right colon ran posterior to the root of small bowel mesentery. The transverse colon then extended to the left upper abdomen to continue as a splenic flexure and descending colon. The transverse colon was completely obstructed by thick fibrotic circular bands arising from the mesenteric root. The colon proximal to the obstruction was massively dilated, as well as the entire small bowel (Figure 3). The small bowel mesenteric root was dissected off the retroperitoneal attachment, and the constricting bands around the transverse colon lysed, thus releasing the obstruction. The liberated colon was left in place without resection or antemesenteric transposition. Schematic illustration of the anatomical situation is depicted in Figure 4.

## 3. Postoperative Course

After the surgery, the patient's postoperative course was complicated by a prolonged paralytic ileus. She was placed on Total Parenteral Nutrition for ten days. Her condition gradually improved, ileus resolved, and she was discharged on the 14th postoperative day. She completed her pregnancy uneventfully and gave birth to a healthy child. Now, she is two years after the surgery and remains completely asymptomatic.

## 4. Discussion

Midgut rotation occurs in two stages. During the first stage, the midgut begins its counterclockwise rotation and herniates through the umbilical cord. In the course of the second stage, the midgut normally completes its rotation and reduces back into the abdominal cavity. Developmental abnormalities of this process may occur at any stage of this process with midgut volvulus being the most common [1]. Reverse intestinal rotation is the rarest form of intestinal rotation and fixation anomalies. It comprises about 4% of all malrotation cases [2]. Reversed malrotation occurs in the second stage. As proposed by Estrada, it takes place when the postarterial bowel segment reduces back into the abdomen first; thus, the transverse colon is brought behind the duodenum and the Superior Mesenteric Artery [3]. Reverse intestinal rotation most commonly presents in infancy or early adulthood with intestinal obstruction and volvulus of nonfixed cecum and colonic obstruction by superior mesenteric vessels [4]. Although intestinal malrotation in adulthood is an extremely uncommon cause of bowel obstruction, several case reports have been reported [5]. Some malrotation cases of midgut volvulus have been reported in pregnant patients as well [6–8]. In 1954, Davis et al. reviewed thirty-one cases of reversed intestinal rotation previously recorded in the literature [9]. They divided reversed rotation into three types:

1. obstruction of the transverse colon due to compression by the superior mesenteric vessels;
2. volvulus of the mobile right colon or the entire midgut;
3. duodenal-jejunal obstruction.

Our case represents the first type of reversed rotation. In 1927, Donald described a case of reversed rotation in a pregnant woman. In his paper, the woman had volvulus of small and large bowel [10]. To the best of our knowledge, this is the first report of reversed rotation, causing large bowel obstruction in a pregnant woman. Intestinal obstruction in pregnancy is uncommon, but when unrecognized in a timely fashion, it is associated with significant maternal and fetal mortality.

Here, we present a rare case of reverse intestinal malrotation in a young pregnant woman. The clinical recognition of the bowel obstruction was delayed in her case due to natural disinclination to perform potentially teratogenic radiological investigations in pregnancy and nonspecific nature of her complaints, mimicking more common hyperemesis gravidarum. She was taken to the operating room based on clinical and radiological ground with suspected mechanical bowel obstruction. The surgical exploration was challenging because of distended bowel and gravid uterus, obscuring lower abdomen and pelvis. After the malrotation was identified and obstructed malpositioned colon was found behind the small bowel mesentery, we performed a simple release of the constricting bands by dissecting small bowel mesentery off its retroperitoneal attachment. It was not possible to perform antemesenteric reposition of the transverse colon because it was almost fused to the posterior abdominal wall with a short mesentery. We considered right colectomy in order to liberate the large bowel from its unfavorable retromesenteric location, but, given the patient general condition, emergency nature of the procedure during pregnancy, distended bowel, and poor nutritional status due to relatively long standing bowel obstruction, we opted for simple lysis of constricting bands and the colon was left in situ. This operative approach worked well for the patient with resolution of the acute obstruction and no further events during followup. Simple release of constricting bands without antemesenteric transposition seems to be the safest option in this situation.
